# *Mycobacterium tuberculosis* Complex Enhances Susceptibility of CD4 T Cells to HIV through a TLR2-Mediated Pathway

**DOI:** 10.1371/journal.pone.0041093

**Published:** 2012-07-23

**Authors:** Seema M. Thayil, Ya-Chi Ho, Robert C. Bollinger, Joel N. Blankson, Robert F. Siliciano, Petros C. Karakousis, Kathleen R. Page

**Affiliations:** 1 Department of Medicine, Johns Hopkins University School of Medicine, Baltimore, Maryland, United States of America; 2 Howard Hughes Medical Institute, Johns Hopkins University School of Medicine, Baltimore, Maryland, United States of America; 3 Department of International Health, Johns Hopkins Bloomberg School of Public Health, Baltimore, Maryland, United States of America; Barcelona University Hospital, Spain

## Abstract

Among HIV-infected individuals, co-infection with *Mycobacterium tuberculosis* is associated with faster progression to AIDS. We investigated the hypothesis that *M. bovis* BCG and *M. tuberculosis* (Mtb complex) could enhance susceptibility of CD4+ cells to HIV infection. Peripheral blood mononuclear cells (PBMCs) collected from healthy donors were stimulated with *M. bovis* BCG, *M. tuberculosis* CDC1551 and *M. smegmatis* MC^2^155, and stimulated CD4+ cells were infected with R5-and X4-tropic single replication-competent pseudovirus. CD4+ cells stimulated with Mtb complex showed enhanced infection with R5- and X4-tropic HIV, compared to unstimulated cells or cells stimulated with *M. smegmatis* (p<0.01). Treatment with TLR2 siRNA reversed the increased susceptibility of CD4+ cells with R5- and X4-tropic virus induced by Mtb complex. These findings suggest that TB infection and/or BCG vaccination may be a risk factor for HIV acquisition.

## Introduction

Chronic immune activation is central to the pathogenesis of HIV. Activation of CD4+ T cells, macrophages, and dendritic cells enhances and promotes viral replication [1], [2], [3]. In the primate model, natural SIV hosts that do not develop pathogenic immunodeficiency have low levels of generalized immune activation compared to pathogenic SIV infections [4], [5]. In humans, systemic markers of immune activation are associated with higher HIV viral loads and faster progression to AIDS [6]. Variations in immune activation among HIV-infected individuals may result from several factors, such as host genetics, microbial translocation, malnutrition, and co-infection with other pathogens [7], [8]. For example, HIV co-infection with pathogens that non-specifically activate host immunity, such as *P. falciparum*, helminths, HSV, and *M. tuberculosis*, has been associated with enhanced virological replication, which could lead to more efficient HIV transmission and faster progression to AIDS [7], [9].

Among HIV-uninfected individuals, immune activation can also enhance HIV susceptibility following exposure to HIV. Non-ulcerative sexually transmitted diseases, such as asymptomatic incident HSV infections, increase the risk of HIV acquisition via local recruitment and activation of CD4+ T cells and macrophages, which increase the number of target cells for HIV entry [10], [11]. In addition to local inflammation, some sexually transmitted infections, such as chronic asymptomatic HSV, are associated with systemic immune activation [12]. While the effect of systemic immune activation on HIV susceptibility has not been firmly established, there is evidence that factors other than local inflammation influence the immunologic milieu at mucosal sites of HIV exposure. For example, the genital tracts of Kenyan women have more activated CD4+ T cells compared to women from San Francisco, independent of genital co-infections or behavioral factors that may influence local genital inflammation [13]. In addition, systemic immunological profiles correlate with natural resistance to HIV, as low levels of peripheral T cell activation are found in individuals who remain uninfected despite frequent exposure to HIV [14], [15]. The elevation of systemic immune activation markers found in HIV-uninfected Africans and Asians compared to Europeans has been attributed to chronic exposure to endemic infections, and may be one of the factors driving regional disparities in HIV rates around the world [16], [17], [18].

Among potential exposures in regions with high HIV rates, *M. tuberculosis* and *M. bovis* BCG (a live attenuated vaccine) are exceedingly common and elicit potent systemic immune responses that may influence HIV infectiousness. It is estimated that worldwide over 2 billion individuals have latent infection with *M. tuberculosis* and almost 10 million new cases of active tuberculosis (TB) occur each year. Despite controversy regarding the efficacy of the BCG vaccine, approximately 100 million doses are given to infants each year. The transcriptional signatures of whole blood obtained from patients infected with *M. tuberculosis* reflect systemic immune activation in both patients with active TB and asymptomatic individuals with latent infection [19], [20]. BCG vaccination can also activate systemic immune pathways associated with T cell activation which persist even after clearance of the bacteria, enhance immune responses to unrelated pathogens in infants, and modulate mucosal immunity [21], [22], [23], [24]. Among HIV-infected individuals, co-infection with *M. tuberculosis* is associated with increased viral load and faster progression to AIDS [25].

Although the effect of TB on HIV pathogenesis has been characterized previously, the impact of systemic immune activation by mycobacterial infections on susceptibility to HIV infection among uninfected exposed individuals is not known. We hypothesized that immune activation by *M. tuberculosis* or *M. bovis* BCG, the two most common mycobacterial exposures worldwide, increases susceptibility to HIV infection. Using a single-cycle infection assay, we examined susceptibility to HIV infection in peripheral blood mononuclear cells (PBMC) stimulated with Mtb complex and identified immune pathways associated with enhanced susceptibility to HIV.

## Results

### *M. bovis* BCG and M. Tuberculosis (Mtb complex) Enhance Infection of CD4+ T Cells with R5-tropic and X4-tropic HIV

**Figure 1:**
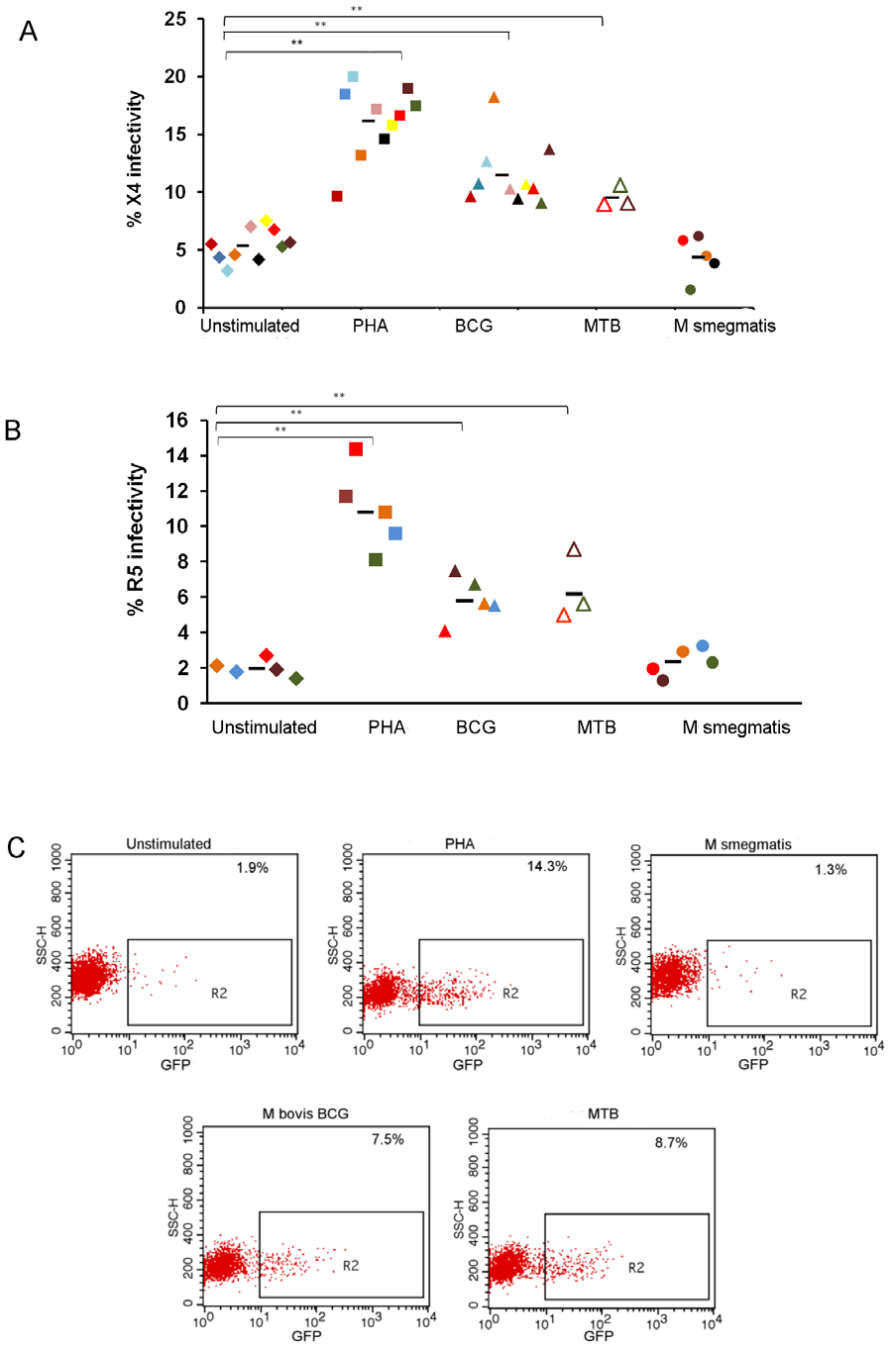
*M. tuberculosis* complex increases susceptibility to HIV infectivity. The percentage of unstimulated CD4+ cells and CD4+ cells stimulated with PHA (50 µg/ml), *M. bovis* BCG (Copehnhagen), *M. tuberculosis* (CDC1551), *M. smegmatis* (MC^2^155) from individual subjects infected with X4-tropic pseudovirus (A) or R5-tropic pseudovirus (B). Results from individual donors are color-coded. Statistical analysis was performed using student T test (** p<0.005). Flow cytometry analysis of the representative CD4+ cells after infection with GFP-expressing pseudovirus (C).

In order to assess whether exposure to mycobacteria affects HIV susceptibility *ex vivo,* we stimulated PBMC from healthy individuals to Mtb complex for 72 hours and subsequently infected the isolated CD4+ cells with an X4- or R5- tropic HIV pseudovirus. CD4+ cells exposed to *Mtb* complex had a significantly (p<0.005) higher HIV infectivity rate with X4-tropic (Fig. 1A) as well as R5-tropic virus (Fig. 1B) relative to that of unstimulated cells and to cells exposed to the nonpathogenic mycobacterium *M. smegmatis*, in which HIV infectivity was similar to that of unstimulated cells. The percentage of HIV-infected cells was comparable following stimulation with *M. bovis* BCG, a known immunomodulator, and with *M. tuberculosis* at the same MOI (Fig. 1C; p = 0.3).

### Exposure to Mtb Complex does not Alter Expression of CCR5 and CXCR4 Co-receptors in CD4+ T Cells

**Figure 2:**
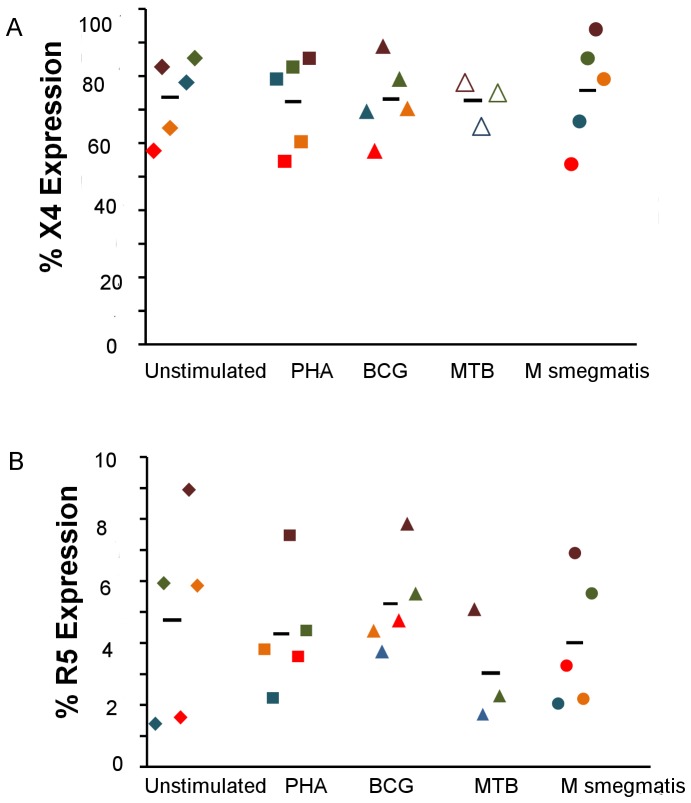
Increase in HIV infectivity is not co-receptor mediated. Surface expression of co-receptor CXCR4 on unstimulated CD4+ cells, CD4+ cells stimulated with phyohemaglutinin (50 mg/ml), *M. bovis* BCG (Copenhagen), *M. tuberculosis* (CDC1551) and *M. smegmatis* (MC^2^155), respectively, at the time of infection with pseudovirus (A). Surface expression of co-receptor CCR5 on CD4+ cells from five subjects after stimulation (B).

To evaluate whether the enhanced infectivity observed in CD4+ cells exposed to Mtb complex was due to modulation of the co-receptors CCR5 and CXC4, we measured the expression of CCR5 and CXCR4 in CD4+ cells exposed to mycobacteria. CCR5 receptors were expressed on 0–5% of CD4+ cells in all experimental and control groups (Fig. 2a). Mean fluorescence intensity values for CCR5 receptor were 366.4+78.1, 436.6+112.3, 336.4+54.7, and 403.4+145.3 for unstimulated CD4+ cells, and those stimulated with *M. bovis* BCG, *M. tuberculosis*, and *M. smegmatis*, respectively. CXCR4 was observed to be expressed on 70–85% of CD4+ cells (Fig. 2b), but expression of this co-receptor did not vary among stimulated and unstimulated cells or between different groups of stimulated cells (p = 0.4). Mean fluorescence intensity values for CXCR4 receptor were 1406.6+132.8, 1540.0+407.3, 1492.6+346.7, and 1403.3+168.5 for unstimulated CD4+ cells, and those stimulated with *M. bovis* BCG, *M. tuberculosis*, and *M. smegmatis*, respectively. We concluded that differential expression of the entry co-receptors CXCR4 and CCR5 does not account for the increased susceptibility to HIV of cells exposed to Mtb complex.

### Increased Immune Activation Markers HLADR and CD38 do not Explain the Increased Susceptibility to HIV Infection Associated with Mtb Complex Stimulation

**Figure 3:**
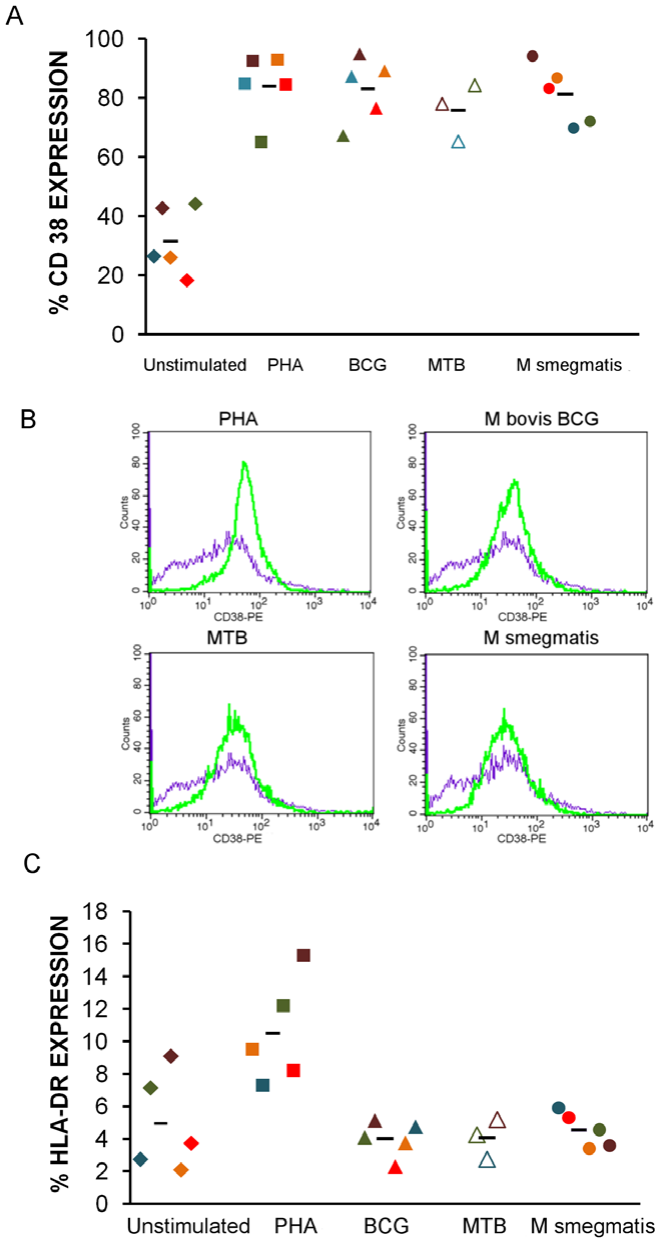
Differential susceptibility to HIV infection is not dependent on increased expression of the immune activation markers CD38 and HLA DR. Cells stimulated with different antigens are activated to a similar extent as evidenced by expression of immune activation marker CD38 (A). Dot plots from unstimulated CD4+ cells and CD4+ cells stimulated with PHA (150 g/ml), *M. bovis* BCG (Copenhagen) and *M. tuberculosis* (CDC1551) depicting the expression of immune activation marker CD38 (B). Cells stimulated with *M. bovis* BCG (Copenhagen) and *M. tuberculosis* (CDC1551) show lower expression of MHC II molecule HLA DR when compared to cells stimulated with *M. smegmatis* MC^2^155 (C) *p<0.05.

Next, we compared the expression of two different immune activation markers on the surface of CD4+ cells stimulated with different mycobacteria (Fig. 3a). As expected, CD38 expression was higher in stimulated cells compared to unstimulated cells (p<0.05), but there was no difference in CD38 expression between CD4+ cells exposed to *M bovis* BCG, *M. tuberculosis*, or *M. smegmatis* (Fig. 3b). CD4+ cells exposed to *M. bovis* BCG and *M. tuberculosis* had diminished expression of HLA DR (p<0.05) when compared to cells exposed to *M. smegmatis* (Fig. 3C).

### Enhanced Expression of TLR2, but not of TLR4, in CD4+ T Cells Exposed to *M. bovis* BCG

**Figure 4:**
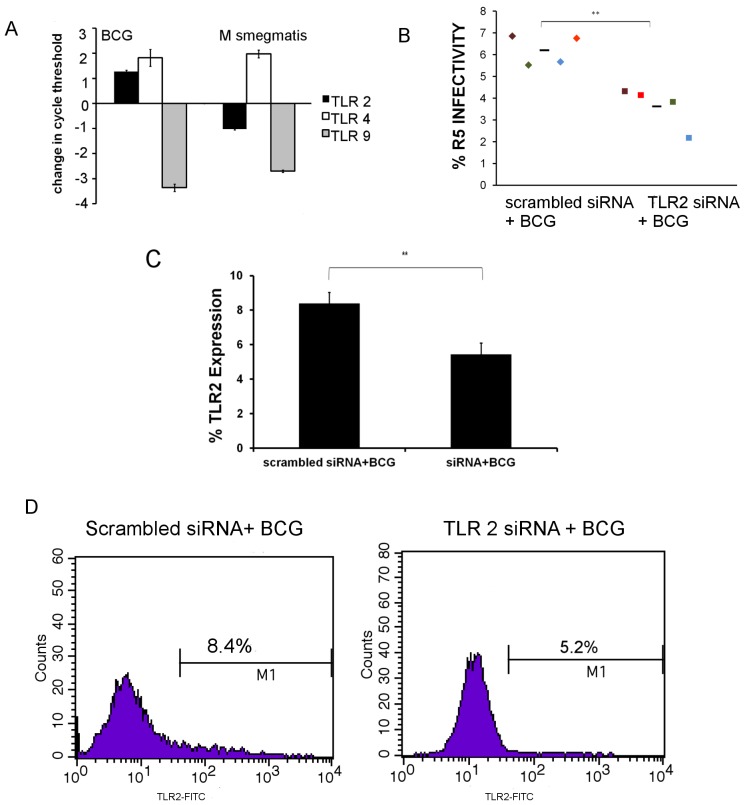
Silencing of TLR2 expression attenuates the increased susceptibility to HIV. Expression of TLR2 increases at the transcription level in cells stimulated with *M. bovis* BCG when compared to cells stimulated with *M. smegmatis* (A). The expression levels of TLR4 and TLR9 do not show a significant difference in cells stimulated with different strains of mycobacteria as evidenced by RT-PCR. Comparison of HIV infectivity of CD4+ cells transfected with scrambled siRNA and subsequently stimulated with *M. bovis* BCG to CD4+ cells transfected with TLR2 siRNA and subsequently stimulated with *M. bovis* BCG (B) **p<0.005. TLR2 expression on CD4+ cells after stimulation with *M. bovis* BCG, *M. tuberculosis*, and *M. smegmatis* compared to TLR2 expression on CD4+ cells transfected with TLR2 siRNA and subsequently stimulated with *M. bovis* BCG (C). The expression showed similar trends for the four samples tested. Dot plots comparing the percentage of surface expression of TLR2 on unstimulated CD4+ cells and CD4+ cells stimulated with *M. bovis* BCG, *M. tuberculosis* (CDC1551), *M. smegmatis* (MC^2^155), and CD4+ cells transfected with TLR2 siRNA and subsequently stimulated with *M. bovis* BCG (D).

Since pathogenic mycobacteria are potent inducers of TLR [26], and TLR-mediated immune activation is associated with HIV replication [27], we evaluated the gene expression levels of TLR2, TLR4, and TLR9 in CD4+ cells exposed to *M. bovis* BCG and *M. smegmatis* by RT-PCR. Although TLR4 expression was higher and TLR9 expression lower in cells exposed to mycobacteria compared to unstimulated cells, there was no difference in expression of TLR4 or TLR9 between cells stimulated with *M. bovis* BCG or *M. smegmatis.* However, gene expression of TLR2 was approximately two-fold higher in cells exposed to *M bovis* BCG than in unstimulated cells (Fig. 4a).

### Increased Susceptibility of CD4+ T Cells Exposed to Mtb Complex is Mediated through a TLR2-dependent Mechanism

In order to determine whether higher expression of TLR2 results in increased HIV susceptibility of *M. bovis* BCG-exposed cells, we silenced TLR2 expression in cells prior to stimulation with *M. bovis* BCG. Relative to *M. bovis* BCG-stimulated CD4+ cells treated with scrambled siRNA, transfection of CD4+ cells with TLR2 siRNA prior to *M. bovis* BCG stimulation decreased infection with R5 virus from 6.2±0.7% to 3.6±1.0% (p = 0.005; Fig. 4b), and with X4 virus from 10.7±0.8% to 7.0±1.0% (p = 0.001; data not shown). Flow cytometry confirmed that prior treatment with TLR2 siRNA reduced TLR2 expression in CD4+ cells stimulated with Mtb complex relative to that of similarly stimulated cells pre-treated with scrambled siRNA with *M. smegmatis* (p<0.005; Figs. 4c and 4d). Therefore, we conclude that the increased susceptibility of CD4+ T cells to HIV infection following Mtb complex exposure is at least partly mediated by TLR2.

## Discussion

In this study, we demonstrate that Mtb complex, but not the nonpathogenic *M. smegmatis,* enhances HIV infection of CD4+ T cells through a TLR2-dependent mechanism. While increased HIV replication has been previously associated with Mtb, this is the first study demonstrating that mycobacterial infections increase CD4 cell susceptibility to HIV infection. Given the magnitude of the TB epidemic worldwide, these findings could have significant public health implications.

We evaluated various immunological pathways that could be associated with increased HIV susceptibility in T cells exposed to *M. tuberculosis* and *M. bovis* BCG, such as increased expression of entry co-receptors (CCR5 and CXCR4), upregulation of immune activation markers HLA DR and CD38, or mediation through TLR pathways known to be modulated by *M. tuberculosis* and *M. bovis* BCG (TLR2, TLR4 and TLR9) [28], [29], [30]. We found that TLR2 was significantly upregulated in T cells exposed to *M. bovis* BCG and that blockade of TLR2 expression attenuated the increased susceptibility to HIV. The role of TLR2 in enhanced HIV susceptibility is consistent with previous studies of HIV and TB pathogenesis. Both *M. tuberculosis* and *M. bovis* BCG are potent inducers of TLR2 [28], [29], [30], [31]. In HIV transgenic mouse spleens, TLR2 signaling enhances HIV replication through trans-activation of HIV long terminal repeats [27], [32]. Signaling through TLR2 has been shown to trigger HIV replication in a variety of human cell lines, including latently infected mast cells and dendritic cells [33], [34]. Co-infection with certain bacteria that stimulate TLR2 can lead to HIV reactivation. For example, periodontal pathogens cause HIV reactivation in a monocyte model of HIV latency via TLR2 activation [35]. HIV transgenic mice infected with *M. tuberculosis* have enhanced viral production, but this effect is not observed in TLR2-deficient HIV mice [36]. Dendritic cells obtained from HIV-infected individuals co-infected with *M. tuberculosis* have increased expression of TLR2 and TLR4, which may promote HIV replication and contribute to the faster disease progression observed in patients with opportunistic infections [37].

In addition to promoting HIV replication in various models of HIV infection, TLR2 has also been implicated in enhancing susceptibility to HIV infection [38]. TLR2 stimulation of Langherhan cells, which are the initial targets of HIV infection following sexual exposure, results in enhanced infection of these cells and subsequent trans-infection of T cells [39]. TLR2 signaling also enhances infection of quiescent naïve and memory CD4+ T cells, which are normally relatively resistant to HIV infection [40]. *N. gonorrhea,* a sexually transmitted pathogen strongly associated with increased HIV risk, enhanced HIV infection of resting CD4+ cells through TLR2 activation [41]. Similar to the mycobacterial effect we observed, the enhanced susceptibility of CD4+ T cells to HIV following exposure to *N. gonorrhea* was reduced upon TLR2 blockade. Recently, Ding and Chang have shown that TLR2 activation promotes HIV infection and nuclear import in resting CD4+ T cells through both T cell activation-dependent and -independent mechanisms [42].

Although previous clinical studies have shown that infection with *M. tuberculosis* or vaccination with *M. bovis* BCG systemically activate TLR2-mediated pathways, more work is needed to determine whether these responses lead to enhanced HIV susceptibility in humans as suggested by our *ex vivo* model [30], [43]. Potential implications of our findings could be significant, especially for regions with a high prevalence of TB and HIV. Our findings may also have implications for TB vaccine development, since current strategies involve genetically modifying the BCG vaccine, developing adjuvants to strengthen protective responses, or using other attenuated mycobacterial species as potential vaccines [28], all of which may activate TLR2-mediated pathways.

There are several important limitations of our model, which require further study to assess the clinical and public health implications of our findings. Direct infection of PBMCs with *M. tuberculosis* complex in our *ex vivo* model most likely overestimates the level of antigenic stimulation resulting from natural infection in the lungs or intradermal vaccination. Further work is needed to determine whether pulmonary TB infection or intradermal BCG vaccination modulates immune responses at sites of HIV exposure, such as the genital and gastrointestinal mucosa, where local flora may drive mucosal immune responses. Although in the current study we have not elucidated the cell type responsible for CD4+ T cell activation, the potential role of different antigen-presenting cells, including macrophages and dendritic cells, deserves further study. One hypothesis is that TLR2-stimulated dendritic cells encountering *M. tuberculosis* or *M. bovis* BCG traffic to lymphoid organs and mucosa-associated lymphoid tissue, where they prime and activate CD4+ T cells. Recent data in a murine model shows dynamic trafficking patterns in dendritic cells exposed to a chronic *M. bovis* BCG granuloma [44]. Despite very low antigenic availability following BCG vaccination, CD4+ T cell priming still was observed as a result of dendritic cell migration from the site of chronic granuloma formation to systemic sites and lymphoid organs. Furthermore, upregulation of TLR2 has been reported in dendritic cells from patients co-infected with TB and HIV [37], [45].

While our findings in an *ex vivo* model of co-infection likely overestimate the impact of dual infection on actual HIV risk, they provide insights about mechanisms of HIV susceptibility that could guide future studies using clinical samples from patients with TB or BCG vaccination. Given the magnitude of the TB epidemic worldwide, even a modest level of enhanced susceptibility to HIV due to *M. tuberculosis* infection or BCG vaccination could have a significant impact at a population level. These results provide initial support for the intriguing possibility that pathogenic mycobacterial infections could partially explain variations in HIV susceptibility worldwide.

## Materials and Methods

### Ethics Statement

Written informed consent was obtained from all subjects prior to entry into the study, which was approved by the Johns Hopkins University School of Medicine Institutional Review Board.

### Subjects

Ten HIV-seronegative healthy individuals (ages 23–54) without prior history of BCG vaccination or TB infection were recruited at Johns Hopkins University School of Medicine. All subjects were confirmed to have negative tuberculin skin tests prior to entry into the study.

### Cell Isolation and Culture

Peripheral blood mononuclear cells (PBMC) were isolated from healthy volunteers and antigenic stimulation of cells was performed by incubating cells with whole bacteria or antigen for 48–72 hours at 37°C with 5% CO_2_. For infectivity assays, unstimulated cells were used as negative control and PHA (50 µg/ml) stimulated cells were used as positive control to ensure that cells were responsive to *in vitro* stimulation.

### Bacterial Strains and Mycobacterial Infection

*M. bovis* BCG (Copenhagen), *M. tuberculosis* CDC1551, and *M. smegmatis* MC^2^155 strains procured from ATCC were used for stimulation of PBMC at an MOI of 1∶4. The bacterial strains were grown to mid-logarithmic phase in 7H9 broth (Difco, Sparks MD) supplemented with 10% oleic acid-albumin-dextrose-catalase (OADC, Difco), glycerol, 0.05% Tween 80 at 37°C on a shaker. At the time of stimulation, bacteria were pelleted and resuspended in complete RPMI and co-cultured with isolated PBMC in complete RPMI supplemented with IL-2 at 37°C for 24–48 hours.

### Single-cycle HIV-1 Infection Assay

After stimulation, the cells were washed with fresh RPMI and CD4+ cells were isolated from stimulated PBMC by negative selection using a CD4+ T cell isolation kit (Miltenyi biotech; GmbH) according to the manufacturer’s instructions. Isolated CD4+ cells were infected with a single-cycle replication-competent, green fluorescent protein (GFP)-expressing proviral construct pseudotyped with CCR5-tropic or CXCR4-tropic envelope (NL43-deltaEnvGFP), as previously described [46]. Briefly, stimulated CD4+ cells were spinoculated for 2 hrs with the virus. The infected cells were incubated at 37°C for 72 hours. Fluorescence emitted by the cells carrying GFP-expressing virus was quantified using Cellquest Pro software (Beckton Dickinson) to calculate percent infectivity.

### Immunoflourescence Staining and Flow Cytometry Analysis

Stimulated CD4+ cells were washed and then stained with directly conjugated antibodies to cell surface markers, including CD4-FITC, CD3-PE, CD38-PE, HLA DR-FITC, CD195–PE, and CD184-FITC (Becton Dickinson, San Jose, CA) and TLR-2 FITC (R&D biosystems). Marker expression analysis was performed using a FACSCalibur flow cytometer (BDIS). A minimum of 10,000 viable lymphocytes was collected for each run.

### Quantitative Reverse-transcription PCR

Total RNA was isolated from 10^6^ to 10^7^ CD4+ stimulated cells using RNeasy Mini Kit (Qiagen). Fluorescently-labeled cDNA was generated using oligod(T) primers and Superscript III (Invitrogen), using fluorescent dyes Cy3 and Cy5 (Amersham). cDNA corresponding to each transcript was subjected to 34 cycles of PCR for quantification using the primers listed in Table S1. The cycle threshold value (C_T_) obtained for each gene of interest (GOI) was normalized with that of GAPDH, a housekeeping gene, in order to obtain the normalized cycle threshold (nC_T_ = (GOI C_T_) – (HKG C_T_)). The change in cycle threshold (ΔCT) was calculated using the following formula: (ΔCT = C_(nCT)_ – S_(nCT)_), where C represents unstimulated cells (negative control) and S represents cells stimulated with mycobacteria or phytohemagglutinin (PHA; positive control). Prior to reverse transcription, control and mutant RNA (10 ng) were treated with RNase-free DNase (Invitrogen) and subjected to 36 cycles of PCR to assure that all DNA had been removed, as assessed by ethidium bromide-stained agarose gel analysis.

### siRNA Transfection

Transfection of monocytes was performed using negative control scrambled small interfering RNA (siRNA) or siRNA targeting TLR-2 (Santa Cruz Biotechnology, Inc.) following the manufacturer’s instructions. Briefly, cells were seeded at 2×10^6^ cells per well in a 6 well plate with reduced siRNA transfection reagent (Santa Cruz Biotechnology Inc.) for 5–8 hours at 37°C with 5% CO_2_. The media were then replaced and the cells were allowed to rest overnight, followed by stimulation with individual strains of mycobacteria. Paired samples were subsequently subject to either immunoflourescence staining with TLR 2 antibody or virus infectivity assays, as described above.

### Statistical Analysis

Statistical significance was determined by students T Test. A p-value of <.05 was considered statistically significant.

## Supporting Information

Table S1 **Primers used for RT-PCR studies.**
(DOCX)
