# Supplementary material for: Mycobacterium tuberculosis Complex Enhances Susceptibility of CD4 T Cells to HIV through a TLR2-Mediated Pathway
Source: PLoS One. 2012 Jul 23;7(7):e41093. doi: 10.1371/journal.pone.0041093 (PMC3402510; doi:10.1371/journal.pone.0041093)
Supplement: Table S1 — Primers used for RT-PCR studies. (DOCX) [file pone.0041093.s001.docx]

|  | **Gene** | **Primer sequence** |
| --- | --- | --- |
| 1 | GAPDH | Forward 5′-GGTGGTCTCCTCTGACTTCAACA-3′  Reverse 5′-GTTGCTGTAGCCAAATTCGTTGT-3′ |
| 2 | TLR2 | Forward 5’-CTGCAAGCTGCGGAAGATAAT-3’  Reverse 5’-GCAGCTCTCAGATTTACCC­AAAA-3’ |
| 3 | TLR4 | Forward 5’-AGAGTTTCCTGCAATGGATCAAG-3’  Reverse 5’-TTATCTGAAGGTGTTGCACATTCC-3’ |
| 4 | TLR9 | Forward 5’- TCTGAAGACTTCAGGCCCAACT-3’  Reverse 5’- TGCACGGTCACCAGGTTGT-3’ |

**Supplementary Table 1: Primers used for RT-PCR studies**
